# Curating Scientific Workflows for Biomolecular Nuclear Magnetic Resonance Spectroscopy

**DOI:** 10.2218/ijdc.v13i1.657

**Published:** 2019-04-19

**Authors:** Douglas Heintz, Michael R. Gryk

**Affiliations:** University of Illinois, Urbana-Champaign; University of Illinois, Urbana-Champaign

## Abstract

This paper describes our recent and ongoing efforts for enhancing the curation of scientific workflows to improve reproducibility and reusability of biomolecular nuclear magnetic resonance (bioNMR) data. Our efforts have focused on both developing a workflow management system, called CONNJUR Workflow Builder (CWB), as well as refactoring our workflow data model to make use of the PREMIS model for digital preservation. This revised workflow management system will be available through the NMRbox cloud-computing platform for bioNMR. In addition, we are implementing a new file structure which bundles the original binary data files along with PREMIS XML records describing the provenance of the data. These are packaged together using a standardized file archive utility. In this manner, the provenance and data curation information is maintained together along with the scientific data. The benefits and limitations of these approaches as well as future directions are discussed.

## Introduction

An acknowledged goal in the field of data curation is to move curation tasks upstream closer to the creation and origination of the data. When considering a scientific study, it is often the case that the digital dataset is not the product of a single, experimental observation. Rather, multiple observations are collected, digitized, normalized, cleaned, and otherwise transformed. Several different and potentially disparate datasets are then analysed in concert along an involved computational pipeline or workflow (Bowers & Ludäscher, 2005).

As the production of computational data through the aforementioned workflows has become increasingly complicated, there has been a growing concern for the lack of a detailed reporting of the workflows along with the intermediate datasets, particularly as these are necessary for the reproducibility and/or repeatability of the computation (Stodden, *et al*., 2016). In the case of such scientific workflows, data curation must be an ongoing process along the entire computational pipeline.

In this paper we discuss efforts to improve the reproducibility of scientific computation by adding curation tasks directly within the construction and execution of the computational workflows. This effort is being conducted within the context of the field of biomolecular nuclear magnetic resonance spectroscopy (bioNMR) using the NMRbox^1^ platform for bioNMR computation. The workflow management system used within NMRbox is CONNJUR Workflow Builder (Fenwick, et al., 2015a). CONNJUR Workflow Builder (CWB) is currently being refactored such that curation metadata will be stored as XML using a hybrid of the PREMIS metadata schema for digital preservation (Denenberg, 2014) and a bioNMR specific metadata schema currently referred to as CONNJUR_ML^2^. The short-term goal is to package this PREMIS XML file together with the scientific dataset (typically a binary file) using standard file archive utilities.

## Biomolecular NMR Spectroscopy

Biomolecular NMR spectroscopy is a biophysical technique which exploits the magnetic moments of the nuclei comprising the matter all around us. A close sibling of Magnetic Resonance Imaging (MRI) which uses this intrinsic magnetism to image human tissue, nuclear magnetism is used in bioNMR studies to explore the structure and dynamics of biological molecules at atomic detail. These studies include determining the three-dimensional structure of proteins and nucleic acids, drug discovery, kinetics and mapping the interfaces of protein-protein and protein-ligand interactions.

The computational workflow for modern bioNMR spectroscopy consists of three phases: spectral reconstruction, the process of converting time domain data into the frequency domain; spectral analysis, including peak identification and resonance assignment; and biophysical characterization, including all subsequent data analysis in which the spectroscopic data is used to draw biophysical inferences (e.g. structure determination) (Ellis, *et al*., 2011; Verdi, *et al*., 2007). The data semantics vary throughout these phases, from primary ‘raw’ data of the nuclear precessions to various levels of derived or interpreted data including resonance frequencies and interatomic distances. This computational workflow uses more than a dozen, academically-developed software tools with many file translation and data cleaning steps along the workflow. Proper curation of the bioNMR workflow is an ongoing challenge affecting data sharing, the archival of research results, and the reproducibility of prior studies (Maciejewski, *et al*., 2017).

**Figure 1.**
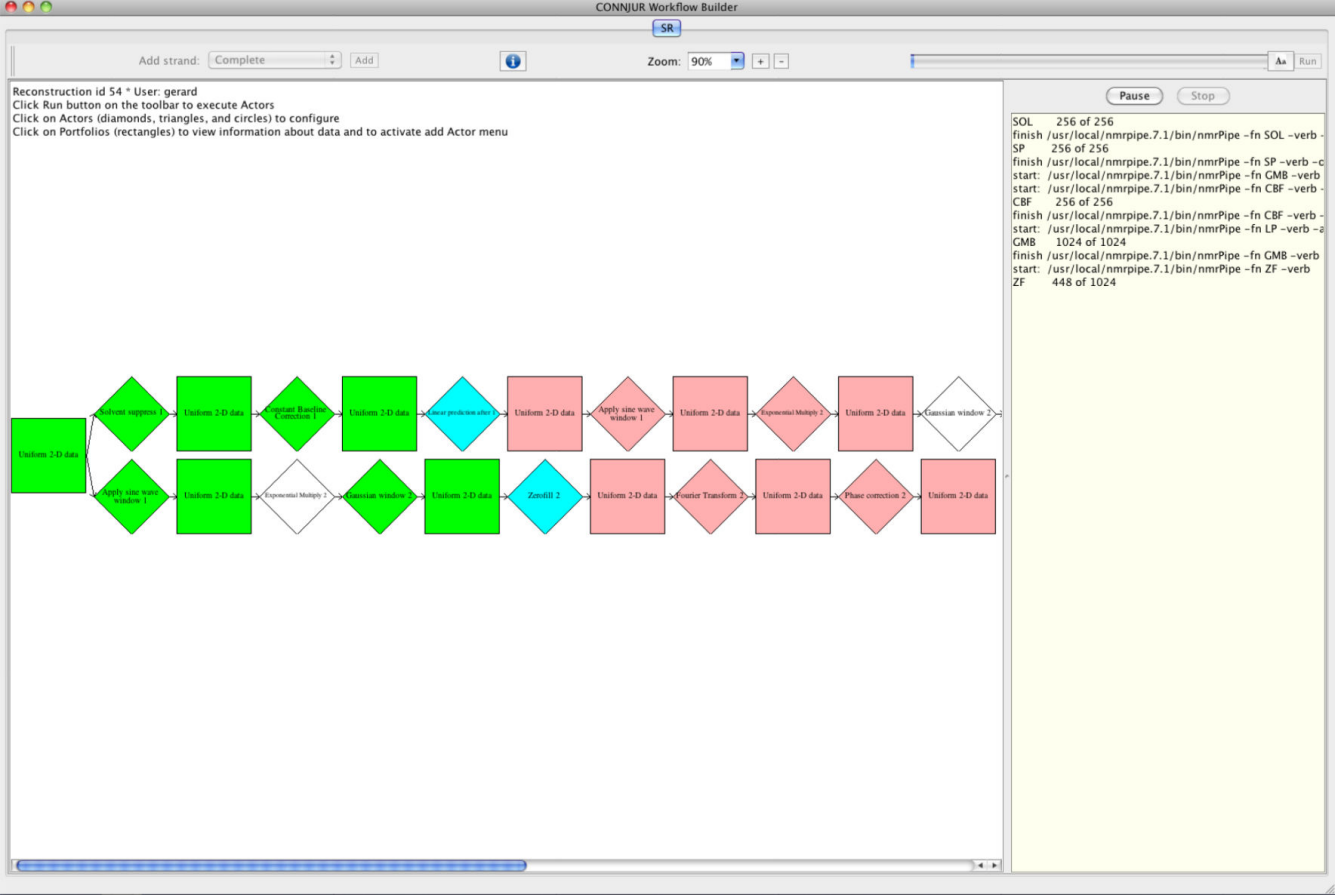
Screenshot of the graphical canvas for CONNJUR Workflow Builder. Squares represent datasets while diamonds represent actors. The above workflow is in the process of being executed. Green objects are those who have completed successively, blue are in progress, and pink actors have yet to be invoked. While actors are being bypassed and not executed in the above workflow.

NMRbox (Maciejewski, et al., 2017) is a recent initiative to foster computational reproducibility for the bioNMR community by (a) establishing an archive of the various software tools for bioNMR and (b) provisioning a virtual machine for bioNMR computation. NMRbox uses CONNJUR for semantic data management within these virtual machines. CONNJUR^3^ is a long-standing project for developing a software integration environment for bioNMR, and currently supports CONNJUR Workflow Builder (Figure 1), a scientific workflow management system for bioNMR spectral reconstruction (Fenwick, et al., 2015a). The benefits of using CWB are that the metadata are stored in a relational database making them available throughout the computational workflow, multiple software tools can easily be interleaved within the workflow, and the workflows can be exported as XML to facilitate reuse and sharing between researchers (Fenwick, et al., 2015a). An example of CWB used for spectral reconstruction is found on our YouTube channel under the title 13C HSQC.

**Figure 2.**
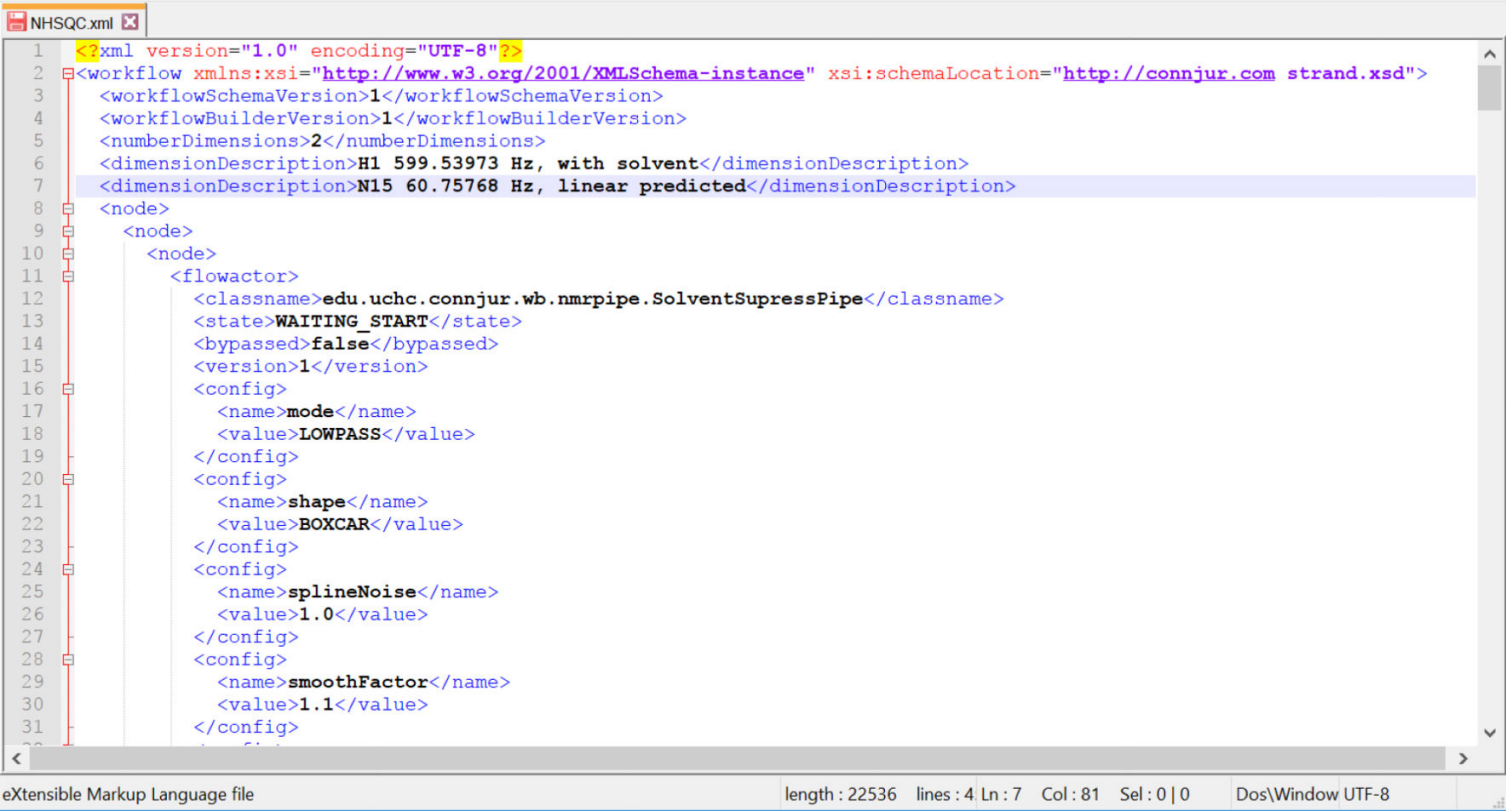
Notepad++ screenshot of original CONNJUR Workflow Builder (CWB) XML schema. While this XML document could be shared between CWB users, the metadata schema was specific for CWB and not intended for broader distribution. It contains information about the software state as well as specific Java classes of software code.

While useful in this context, the data model for CWB is restricted to the context of bioNMR and the implementation details for constructing and executing the workflow itself. As pointed out by Willoughby and Frey (2017), such customization has the drawback that the reuse and sharing enabled by workflow management systems such as CWB is limited in scope to the users of CWB. This can be more readily appreciated by examining the XML output from version 1 of CWB. (Figure 2). The XML for the exported workflow contains information about the Java classes required to configure and execute the workflow within the CWB program. With appropriate knowledge of the operation of CWB and the XML itself, it should be possible to translate such a workflow into a more generic description amenable to other workflow management systems. However, this knowledge requirement is quite extensive and has prompted us to examine a more generic workflow representation which is both useful for the bioNMR community it serves, as well as the broader audiences of workflows, provenance and digital curation. The broad XML architecture chosen for this purpose is that of PREMIS, a general framework used by the digital preservation community.

## PREMIS

Maintained by the Library of Congress, Preservation Metadata: Implementation Strategies (PREMIS) has been developed by the archival and library communities to provide digital preservation systems with a framework to build reliable systems for sustainable information stewardship (Deneberg, 2014). With the release of version 3 of PREMIS, it is possible to embed custom structural, descriptive and administrative metadata within a PREMIS XML record. We have developed a bioNMR XML for spectral reconstruction which is currently made available on GitHub. Called CONNJUR_ML, this bioNMR XML is intended to provide metadata suitable for the significant_properties field in PREMIS objects, as well as the extension field for PREMIS agents to provide metadata about the software and hardware environments.

Digital preservation is itself a type of workflow, albeit a very general workflow supporting a wide assortment of digital objects. CWB was re-assessed in the context of PREMIS as it became clear that the panoply of workflow components for spectral reconstruction are all variations on the data cleaning, data translation, data normalization and data transformation operations typical for digital preservation workflows. The important metadata being captured by CWB throughout the workflow are structural and administrative metadata important for both describing how the component processing steps were configured, and also for identifying classification properties of the intermediate datasets. Willoughby and Frey (2017) call attention to the importance of the intermediate data generated during a scientific workflow. In the case of bioNMR, the intermediate datasets can be quite large, often gigabytes in size. We have found that a detailed metadata record can be a suitable surrogate to storing the actual intermediate files.

## CONNJUR Workflow Builder

The data model for CONNJUR Workflow Builder is being refactored to support the more general concepts provided by PREMIS, as well as the bioNMR-specific classifications of the bioNMR data. The next version of CWB will import and export spectral reconstruction workflows as PREMIS digital preservation records, widening the audience for data sharing and reuse. As part of its integration within the NMRbox platform, CONNJUR/PREMIS workflows will also be translated into NMR-STAR, the file format supported by the primary biomolecular NMR data repository, the BioMagResBank^4^ (Ulrich, *et al*., 2008). By supporting linked digital preservation events, the PREMIS metadata standard provides provenance of what operations were performed on any digital object (in our case, bioNMR datasets), when they were performed, by what human agents and using what software tools - up to and including the entire software environment. As part of the NMRbox platform, this entire software environment is archived and will be accessible as a virtual machine for several years to come.

**Figure 3.**
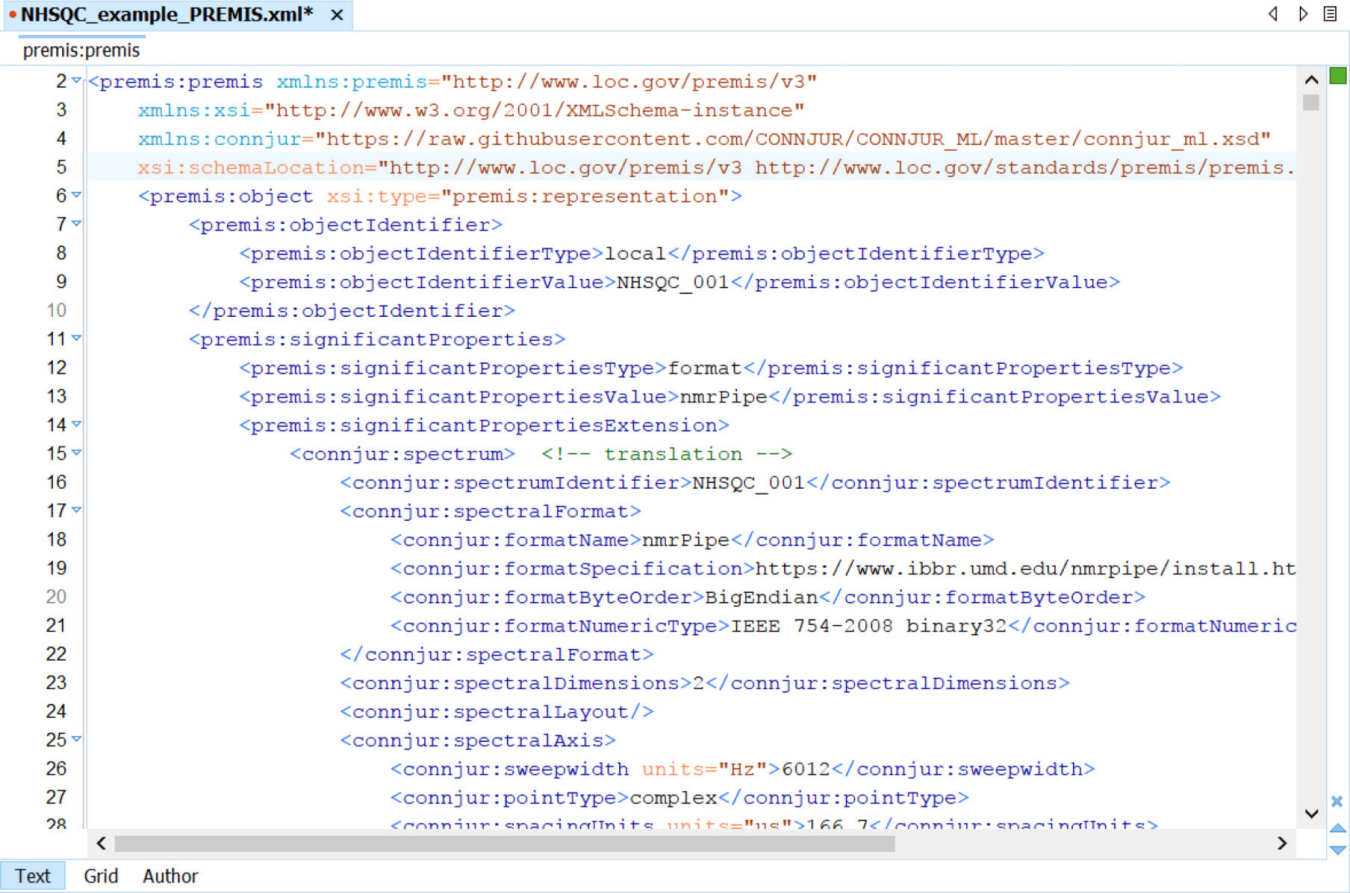
Oxygen screenshot of PREMIS XML record with CONNJUR_ML metadata embedded within the ‘significantProperties’ PREMIS tag. CONNJUR_ML is an ongoing modelling task and the XML can be found on GitHub.

Figure 3 shows a snippet of a CWB metadata record using PREMIS in conjunction with CONNJUR_ML (the actual metadata record for this workflow is over 1000 lines long). PREMIS is used to define the intellectual objects represented by each intermediate dataset along the computational pipeline. As these intermediates are only intended to be stored local to the execution environment, they are given local identifier types and values.

Each intermediate dataset is manifested (perhaps only transiently) in a file format which can be interpreted by the underlying software tool which will process the data. The format of the data is recorded using the PREMIS significant properties tag. All additional structural metadata about the intermediate dataset is contained in the significant properties extension section. This makes use of the bioNMR specific CONNJR_ML to record important features of the intermediates – such as the levels of signal coverage, resolution, as well as various state information regarding the types of transformation which have been applied. This structural metadata can be used as a sort of fingerprint for (a) classifying which types of transformations can be made on which datasets as well as (b) for validating the workflow.

Each subsequent processing step, in which an actor ingests a dataset and produces another, is recorded as a PREMIS event with associated metadata describing and linking the events to the intellectual objects with which they are involved. Additionally, each PREMIS event is associated with a PREMIS agent which may be a human or a computer. For the computational workflows executed by CWB, additional metadata on the software and software versioning is added to the PREMIS agent extension.

## Limitations and Future Directions

Limitations of this approach are related to that of virtual machines themselves. While the digital image of a virtual machine can be stored indefinitely, its operation is dependent on suitable computing hardware and hypervisor software capable of emulating the software environment. Virtual machines are currently ubiquitous and are expected to remain so for years to come; however, future migration to new hardware/software would seem unavoidable.

It is for this reason that many scholars concerned with reproducibility are advocating the use of container technology such as Docker. Docker containers provide a lighter footprint of virtualization and might be expected to remain stable farther into the future than full-fledged VM’s. However, NMRbox currently supports over 100 individual software tools. To package each of these tools within an individual container and have the individual containers all interoperate is a significant task. Nevertheless, this usefulness of containers is being explored by the developers at NMRbox.

In this context of long-term sustainability, one of the added benefits of describing workflows within a digital preservation standard such as PREMIS is that the transformations along the workflow are described using a broader language, making the context and purpose of the workflows accessible to a wider audience. In this sense, our workflow management system is being repurposed as a data curation tool.

Our current metadata model and workflow implementation is for well-structured workflows using primarily mathematical transformations. Additional future directions will be to provide for more general data curation activities and more free-form annotation schemes such as done for spectral analysis using the reproducibility extensions of NMRFAM-Sparky (Fenwick, et al., 2015b)

## Footnotes

1 http://www.nmrbox.org

2 http://www.github/CONNJUR/CONNJUR_ML

3 http://www.connjur.org

4 http://www.bmrb.wisc.edu/
